# Non-Interventional Management of Advanced Pancreatic Neuroendocrine Neoplasms in Patients with von Hippel-Lindau Disease

**DOI:** 10.3390/cancers15061739

**Published:** 2023-03-13

**Authors:** Reut Halperin, Amit Tirosh

**Affiliations:** 1 ENTIRE Endocrine Neoplasia Translational Research Center, Sheba Rd. 2, Ramat Gan 6562601, Israel; 2 Faculty of Medicine, Tel Aviv University, Tel Aviv 6997801, Israel; 3 Division of Endocrinology, Diabetes and Metabolism, Sheba Medical Center, Ramat Gan 5266202, Israel

**Keywords:** pancreatic neuroendocrine neoplasms, von Hippel–Lindau, metastasis, HIF inhibitor, VEGF receptor inhibitor

## Abstract

**Simple Summary:**

Von Hippel–Lindau (VHL) is a hereditary syndrome associated with various tumors in the brain, spine, eyes, kidneys, adrenals, and in the pancreas. Approximately a fifth of patients with VHL develop tumors in the pancreas, and most of these tumors are localized and can be followed or resected in surgery. However, about a tenth of these patients develop metastatic disease which requires treatment with drugs. This review details the various medical interventions available for treating these patients. Such medications target the key factors that are overactive in tumor cells in the context of VHL. Many of them have been tested in clinical studies, and some have been approved recently for clinical use for localized pancreatic tumors in patients with VHL. To assist in managing patients with VHL and metastatic pancreatic tumors, we suggest an algorithm for choosing the optimal medical intervention for patients in this rare scenario.

**Abstract:**

Von Hippel–Lindau (VHL) is a rare autosomal dominant hereditary cancer predisposition syndrome. Patients with VHL have a high risk for developing retinal and central nervous system hemangioblastoma, pheochromocytoma, renal cell carcinoma, and pancreatic neuroendocrine neoplasms (PNEN). About a fifth of patients with VHL will develop PNEN, and only a tenth of them will develop metastatic or unresectable (advanced) PNEN requiring medical intervention. In this review, we performed a literature search for studies, written in English, on the medical interventions for VHL-related localized and advanced PNENs and their clinical outcomes. We detail the various medical interventions for this rare group of patients, including their mode of action and potential efficacy and toxicity. Finally, based on the current literature, we delineate a possible management algorithm for patients with VHL and advanced PNEN. We can conclude that data on the efficacy of various vascular endothelial growth factor (VEGF) receptor inhibitors, and on the efficacy of belzutifan, a novel hypoxia-inducible factor 2 inhibitor, for the management of advanced PNEN in VHL, are scarce. Hence, deduction from the management of sporadic PNEN is required, and is implemented in the proposed management algorithm provided within this review.

## 1. Introduction

Neuroendocrine neoplasms of the pancreas (PNEN) are rare neoplasms, with an estimated annual incidence rate of 0.8 per 100,000 patients in the general population [1]. They are graded according to rate of mitoses and percentage of Ki67, with low-grade comprising G1 (mitoses < 2 per 2 mm and Ki67 < 3%) and G2 (2–20 mitoses per 2 mm, and Ki67 of 3–20%) and high-grade comprising G3 (mitoses > 20 per 2 mm or Ki67 >20%) [2]. About 10–40% of PNENs are functional; that is, they have the potential to cause secretory syndromes such as hypoglycemia due to insulin over-secretion (insulinoma), recurrent gastric ulcers due to gastrin over-secretion (Zollinger–Ellison syndrome) and others [3]. 

Von Hippel–Lindau (VHL) disease is a hereditary cancer predisposition syndrome, inherited in an autosomal dominant trait. VHL disease is caused by a germline DNA alteration in the *VHL* gene, located at 3p25. Patients harboring a germline pathogenic variant in the *VHL* gene have a high risk for developing central nervous system and retinal hemangioblastomas, adrenal pheochromocytoma and extra-adrenal paraganglioma, clear-cell renal cell carcinoma, cysts in the kidneys, pancreas, liver, testicles and broad ligament, endolymphatic sac tumors, and PNENs [4]. The risk for the various manifestations of VHL was found to be associated with the genotype, with patients harboring a non-missense pathogenic variants leading to a truncated VHL protein having a very low risk of developing pheochromocytoma [5]. However, there are no prospective studies supporting genotype-based follow-up algorithms or risk stratification. Thus, the proposed diagnostic, follow-up and management algorithms that currently exist are used broadly for all patients with VHL [4,6,7].

In VHL, PNEN prevalence ranges between 1 and 17% in the various cohorts [4,6,8,9,10,11] with age at diagnosis significantly younger in VHL-related PNEN (vPNEN) compared with sporadic PNEN (sPNEN) [12,13]. vPNENs are more frequently multifocal and very rarely functional [13,14,15]. vPNENs usually follow a more benign course with lower grade, G1\G2 in most cases [16], are less prone to metastasize than sporadic disease, and the patients are at a lower mortality risk than those harboring sPNENs [13,17]. In the European-American-Asian-VHL-PNEN-registry [18], comprising 273 patients with vPNEN, metastatic disease was found in 20% of cases, while a much lower risk was reported in other cohorts, ranging between 4.5 and 8.3% of cases [10,16]. Risk factors for metastatic disease in vPNEN include lesion diameter greater than 3 cm, a short lesion volume doubling time (<500 days), and presence of a missense *VHL* gene pathogenic variant or a pathogenic variant located in exon 3 [10,16,18,19].

Management guidelines differ between sPNEN and vPNEN. For instance, considering the high pretest probability for PNEN in patients with VHL, the current management guidelines for vPNEN suggest against routine biopsy of a pancreatic lesion when it has typical radiological characteristics (enhancing on the arterial phase of contrast injection) [20], while in the diagnostic process and management of patients with sporadic disease, histopathological diagnosis is the rule, except for rare and unique clinical situations [3]. 

The term “advanced PNEN” refers to lesions that cannot be fully resected, either because there are distant metastases, or due to localized involvement of adjacent structures that preclude surgical removal. Considering the lack of robust evidence on the rare advanced vPNEN, the current management guidelines for vPNEN suggest extrapolating the management of advanced vPNEN from sPNEN [3,20]. However, the unique pathophysiology of VHL, of an unrestrained activity of hypoxia-inducible factor (HIF) in the absence of a functional VHL protein, opens-up new systemic pharmacological treatment options [4]. In this review, we will focus on systemic pharmacological treatments for metastatic or locally advanced vPNEN and introduce a possible treatment algorithm.

## 2. VHL-Mechanism-Based Interventions for vPNEN

In VHL, the *VHL* tumor suppressor gene is altered, leading to reduced expression of a functional VHL protein (pVHL). Normally, pVHL has a key role in the ubiquitination of HIF1a and HIF2a under normal oxygen levels (normoxia), leading to their degradation. Under hypoxia conditions, HIFs act as transcription factors which drive cell proliferation, angiogenesis via vascular endothelial growth factor (VEGF) upregulation, and erythropoiesis by upregulating the *EPO* gene, encoding erythropoietin [4]. In VHL disease, the abnormally low levels of functional pVHL lead to a state called pseudohypoxia, in which HIF1a and HIF2a are not degraded and are active in a non-regulated manner [21]. Unrestrained HIF activity leads to the generation of highly vascular neoplasms and cysts in the organs involved in VHL. Considering this clear pathogenic mechanism driving tumorigenesis in patients with VHL, it is clear why both HIF and VEGF are the main targets for medical intervention, by using HIF2 and VEGF/VEGF receptor inhibitors, as detailed below and in Table 1.

### 2.1. Tyrosine Kinase Inhibitors (TKIs) with Vascular Endothelial Growth Factor (VEGF) Receptor Inhibition Ability

VEGF is a key factor in pseudohypoxic states, and specifically in the pseudohypoxia induced by pVHL deficiency. The VEGF receptor (VEGFR) is a tyrosine kinase. The binding of VEGF to the VEGFR activates it, and initiates various intracellular signaling pathways that eventually promote angiogenesis, a critical factor in the neoplastic process and in neoplasia development, progression and spread [26].

There are several clinical trials involving VEGF/VEGFR inhibitors in patients with VHL, as described below. In the largest retrospective study [27], 32 patients with VHL, including 21 patients harboring pancreatic neoplasm(s) or cyst(s), were treated with various TKIs: sunitinib (n = 12, eight patients with pancreatic involvement), sorafenib (n = 11, eight patients with pancreatic involvement), axitinib (n = 6, four patients with pancreatic involvement) or pazopanib (n = 3, one patient with pancreatic involvement). Of a total of 15 evaluable pancreatic lesions, 11 were stable and four showed partial response (one treated with sunitinib, one treated with pazopanib, and two treated with sorafenib). However, as the type of pancreatic lesions (cyst vs. PNEN) were not detailed, one cannot conclude whether there was a clinically significant response of PNEN to treatment with TKIs. 

#### 2.1.1. Sunitinib

Sunitinib is a multi-kinase inhibitor with a VEGF receptor inhibitory function. In sPNEN, a phase three trial [28] including 171 patients with well-differentiated PNEN, oral sunitinib (37.5 mg one daily) demonstrated superior progression-free survival (PFS) vs. placebo (11.4 vs. 5.5 months, respectively), with a hazard ratio for disease progression or death of 0.42 (95% confidence interval (CI), 0.26 to 0.66, *p* < 0.001) and benefit in overall survival (OS, hazard ratio of 0.41 for sunitinib, 95% confidence interval 0.19–0.89, *p* = 0.02) compared with placebo. The most common adverse events (>30%) reported for sunitinib included diarrhea (59%), nausea (45%), weakness (34%), vomiting (34%), and fatigue (32%). Neutropenia (12%) and hypertension (10%) were the most common grade 3–4 adverse events in the sunitinib group.

In vPNEN, sunitinib was tested in a pilot study assessing its efficacy and toxicity in patients with VHL [22]. Fifteen patients were enrolled, seven with PNENs that were up to 3 cm in size. Treatment included sunitinib 50 mg daily for 28 days followed by a 14-day break for up to four cycles. Nine out of the 15 patients completed four cycles, and ten had the sunitinib dose reduced. All five patients with evaluable PNEN had stable disease at the end of follow-up. Other available literature includes several case reports of patients with vPNEN treated with sunitinib and demonstrating either partial response [29,30,31] or stable disease [32].

#### 2.1.2. Vandetanib

Vandetanib is an orally administered TKI, targeting RET and VEGFR [33]. Vandetanib is used for locally advanced or metastatic medullary thyroid carcinoma [34]. In this patient population, the main adverse events included diarrhea (56%, 11% grade ≥3), rash (45%), nausea (33%), and hypertension (32%). In patients with VHL, vandetanib was evaluated in a phase II single arm study (n = 37), that included two patients with measurable pancreatic lesions [23]. All patients received treatment with vandetanib 300 mg/day for 28 days. During the short study period, both patients with pancreatic lesions had stable disease. However, four (10.8%) patients did not complete the study due to toxicity.

#### 2.1.3. Pazopanib

Pazopanib is an oral multitargeted tyrosine kinase inhibitor, inhibiting VEGFR, fibroblast-derived growth factor receptors, platelet-derived growth factor receptors, and cKit [35]. In patients with sporadic neuroendocrine neoplasia, pazopanib was evaluated in several phase II studies, all including patients with sPNEN. In a study by Phan et al. [36], seven (22%) of 32 patients with sPNEN (72% G1, 28% G2) achieved partial responses, with an overall objective response of 21.9% (95% CI 11.0–38.8). The most common adverse events included fatigue (67%), diarrhea (58%), hypertension (42%), and elevated plasma transaminases levels (27% and 38%), with 12% and 8% of the patients reporting grade 3 hypertension and fatigue, respectively. 

In the context of VHL, Jonasch et al. reported a phase II trial in which pazopanib was assessed for the treatment of VHL-related renal cell carcinoma in 32 patients [24]. The cohort included nine patients with 17 pancreatic lesions. Nine (53%) pancreatic lesions showed partial response to treatment with pazopanib (800 mg daily for 24 weeks), and the other eight lesions remained stable. Overall, seven (21%) patients of the entire cohort discontinued treatment due to grade 3 toxicity (four patients, 13%) or repeated grade 1–2 toxicity (three patients, 10%). 

### 2.2. Hypoxia-Inducible Factors (HIF) Inhibitors

#### Belzutifan

Belzutifan (Welireg^®^) is a recently developed HIF2a inhibitor, uniquely designed to counteract the constitutive activation of HIF in VHL disease-related neoplasms. Belzutifan was studied in a phase II clinical trial [25] designed to assess the efficacy and safety of belzutifan in patients with VHL. The primary end point of this single arm study was the response of non-metastatic RCC to 120 mg oral daily dose of belzutifan. Sixty-one patients with VHL were enrolled, of whom 22 (36%) had vPNEN. vPNEN response was observed in 20 out of 22 (91%) vPNEN, including complete response in three patients. The median time to response for vPNEN was 5.5 months. Of note, treatment resulted in response of the more common VHL manifestations, with an objective response rate of 49% and 30% for RCC and central nervous system hemangioblastomas, respectively. The benefit was reinforced by the marked reduction in the interventions required for VHL-related neoplasm indications following the initiation of belzutifan. 

While the treatment was overall well tolerated all patients had at least one treatment related side effect, most commonly anemia (90%, grade 3 in 8% of patients), fatigue (66%, grade 3 in 5% of patients), headache (41%, 0% grade 3) and dizziness (39%, 0% grade 3). Overall, 7 out of 61 patients discontinued treatment and 15 had dose reduction. One patient died, due to fentanyl intoxication. 

The role of belzutifan for advanced sPNEN and vPNEN, and assessment of belzutifan for localized vPNEN, as a primary endpoint, is currently conducted in the LITESPARK-015 trial. This trial is also designed to assess belzutifan efficacy on VHL-related pheochromocytomas and paragangliomas, a VHL manifestation that was not previous evaluated under belzutifan treatment [37].

## 3. Non-VHL-Mechanism-Related Treatments for vPNEN

While vPNENs have unique biological and clinical characteristics compared with sPNENs, in the absence of prospective studies suggesting interventions for advanced vPNEN, the current management recommendations suggest using the interventions based on evidence from sPNEN studies. In this part of the review, we will outline the mainstay pharmacological treatment for advanced well-differentiated sPNEN that can be extrapolated to vPNEN (Table 2).

### 3.1. Somatostatin Analogues (SSA)

Two randomized controlled trials tested the effect of SSA on the progression of well-differentiated gastro-entero-pancreatic (GEP) neuroendocrine neoplasms. The PROMID trial included patients with advanced G1–2 midgut NETs treated with octreotide LAR (intramuscular 30 mg/month, 42 patients) vs. placebo (43 patients) until disease progression [38]. There were significantly less events of disease progression in the octreotide vs. control arms, with 67% reduced risk of progression (95% CI, 0.19 to 0.55, *p* <0.001). Median PFS was 14 months for the octreotide LAR arm (95% CI, 11 to 28.8 months) and 6 months in the control arm (95% CI, 3.7 to 9.4 months). Adverse events occurred more often in the octreotide group, and included diarrhea, flatulence, and cholelithiasis. However, the rate of serious adverse events was similar between groups. 

The CLARINET trial [39] evaluated the effect of lanreotide autogel (depot) treatment (subcutaneous 120 mg/month, 101 patients) compared with placebo (103 patients) in advanced G1 or G2 (Ki67 < 10%) non-functioning GEP-NET. The disease was stable in 96% of the patients at enrollment, and treatment duration was 96 weeks. Patients treated with lanreotide autogel had a significantly prolonged PFS vs. the placebo arm (median not reached vs. 18 months, respectively, *p* <0.0001) with 53% reduced risk of disease progression or death (95% CI, 0.30 to 0.73). At 24 months, 65% in the lanreotide arm (95% CI, 54.0 to 74.1) and 33% in the control arm (95% CI, 23.0 to 43.3) remained progression free. However, in the subgroup analysis including only patients harboring PNEN, no statistically significant difference was found between the treatment and the placebo arms (95% CI, 0.32 to 1.04). 

Both the PROMID and the CLARINET trials included crossover of patients from the placebo to the treatment arm. Hence, overall survival was not significantly different. Adverse events were observed in 50% of patients in the lanreotide arm, including diarrhea (26%), abdominal pain (14%), cholelithiasis (10%), and hyperglycemia (5%), with lanreotide-related serious adverse events reported in only 3% of patients (including one case each of hyperglycemia, diabetes mellitus, nausea, vomiting, abdominal pain, biliary fistula, and cholelithiasis).

### 3.2. SSA-Base-Based Peptide Receptor Radionuclide Therapy (PRRT)

Commonly used PRRT is based on the coupling of the beta and gamma emitter Lutetium-177 (^177^Lu) radionuclide to a somatostatin analogue such as DOTATATE (^177^Lu-DOTATATE, ^177^Lu-DOTA^0^-Tyr^3^–octreotate). Treatment is based on the expression of somatostatin receptors on the tumor cells, evaluated by avidity of the lesions to Galium-68 (^68^Ga)-DOTATATE on positron emitting tomography/computerized tomography (PET/CT) [43].

The NETTER-1 phase III study enrolled patients with advanced G1/G2 midgut NETs that showed progression under up to 30 mg of octreotide every 3 weeks [41]. Patients were randomized to treatment with either ^177^Lu-DOTATATE every two months for up to four treatments combined with monthly 30 mg octreotide LAR (111 patients) or to treatment with high-dose (60 mg) monthly octreotide LAR (110 patients). At 20 months, the rate of PFS was 65% in the ^177^Lu-DOTATATE group (95% CI, 50.0 to 76.8) vs. 11% in the control group (95% CI, 3.5 to 23.0), with a statistically significant 79% risk reduction for disease progression or death in the ^177^Lu-DOTATATE group (95% CI, 0.13 to 0.33, *p* < 0.001). Overall survival in the interim analysis was significantly higher in the ^177^Lu-DOTATATE vs. control group, with a 60% reduced hazard ratio (*p* = 0.004). In terms of safety, 77% of patients in the ^177^Lu-DOTATATE received all four treatment cycles. Drug-related adverse events were observed in 86% of the patients in the ^177^Lu-DOTATATE group and in 31% of the control group and were most often mild (grade 1–2) and gastrointestinal-related (59% nausea, 47% vomiting, and 40% fatigue). Grade 3–4 adverse events in the ^177^Lu-DOTATATE group included lymphopenia (9%), vomiting (7%), and thrombocytopenia (2%). It is important to state that nausea and vomiting are mainly attributed to the use of VAMIN-18 for amino acid administration, which is rarely in use these days. Vomiting is rarely cause by the PRRT per se. In the extended NETTER-1 follow-up trial, overall survival after 5 years from enrollment was assessed. However, there was no significant difference between the treatment groups [44].

In PNEN, several retrospective studies suggested that PRRT treatment is efficacious [45], and in fact may show even higher efficacy in PNEN compared with small intestine (formerly termed “mid-gut”) NEN. Brabander et al. reported complete response with PRRT in 5% of PNEN vs. 1% in small intestine NET, partial response in 50% and 30%, and progressive disease in 13% and 9%, respectively, overall demonstrating a higher success rate for PNEN compared with the neoplasm evaluated in the NETTER-1 trial [46]. In addition, retrospective studies demonstrate superiority in PFS for upfront PRRT treatment in advanced PNEN vs. either chemotherapy (temozolomide, cisplatin, oxaliplatin, or fluorouracil) or targeted therapy (everolimus or sunitinib) [47,48].

The long-term toxicity concerns of PRRT involve myelodysplastic syndrome (MDS), acute leukemia, and liver or renal failure. In a large retrospective assessment of PRRT safety, Barbander et al. reported acute leukemia in 4/610 (0.7%) and MDS in 9/610 (1.5%) patients, with no long-term renal or hepatic failure [46].

There are several ongoing randomized clinical trials comparing the efficacy of ^177^Lu-DOTATATE compared with octreotide or to best standards of care (such as everolimus and sunitinib) in advanced G2–3 GEP-NET patients [48].

### 3.3. Mechanistic Target of Rapamycin (mTOR) Inhibitors

Mammalian target of rapamycin (mTOR) is a kinase pathway involved in cell proliferation and angiogenesis. Somatic alterations in genes related to the mTOR pathway are found in PNENs [49,50], and the pathway is a target for antineoplastic intervention in PNEN as well as in RCC.

#### Everolimus

Everolimus is an mTOR inhibitor with proven efficacy for the treatment of RCC, both as monotherapy and in combination therapy [51,52]. The Radiant-3 trial [40] was a phase III randomized controlled trial of 410 patients with advanced G1/G2 sPNEN that showed advancement within 12 months prior to enrollment. Patients were assigned to receive either everolimus (10 mg per day, orally), an mTOR inhibitor, or placebo until disease progression. Dose adjustment was required for 59% of patients in the everolimus arm. Progression-free survival was 11 months for patients treated with everolimus vs. 4.6 months in the control arm (65% risk reduction, 95% CI, 0.27 to 0.45, *p* < 0.001). Progressive disease was found in 14% of patients in the everolimus arm compared with 42% in the placebo arm. Stable disease and objective response were seen in 73% vs. 51% and 5% vs. 2% in the everolimus vs. placebo arm, respectively. Overall survival was not assessed, as 73% of the patients in the placebo arm crossed over to receiving everolimus. The most common adverse events were stomatitis (64%), rash (49%), diarrhea 34%), fatigue (31%), and infections (23%), mostly of grade 1 or 2. Grade 3 and 4 adverse events included anemia (6%), thrombocytopenia (4%), hyperglycemia (5%), stomatitis (7%), and diarrhea (3%). One drug related death in the everolimus arm occurred, due to acute respiratory distress syndrome.

There are no studies designed to test the effect of everolimus treatment in vPNEN. A retrospective study of PNEN in Brazil included two patients with vPNEN; however, treatment effect cannot be assessed as these patients’ outcomes were pooled within a germline-related PNEN including both MEN1 and VHL patients [53].

### 3.4. Chemotherapy

High-grade PNEN is rarely encountered in patients with VHL. That being said, chemotherapy, and specifically capecitabine and temozolomide, is a relevant intervention in patients with well to moderately differentiated PNEN, such as G2 PNEN with KI67 >10%. In these tumors, there is often a weaker expression of somatostatin receptors, thus not enabling effective treatment with somatostatin analogues and/or with the radiolabeled somatostatin analogues derivatives (peptide receptor radionuclide therapy with ^177^Lu-DOTATATE, PRRT). Hence, treatment with chemotherapy vs. mTOR inhibitors and TKIs is often considered. In patients with VHL, this consideration is more complex, since there is a higher chance that patients will require bone-marrow-toxic intervention for other indications. Hence, in a patient with VHL and advanced PNEN, one may consider avoiding chemotherapy or PRRT whenever possible, and prefer TKIs or mTOR inhibitors, to preserve bone marrow function in the long run.

#### Capecitabine and Temozolomide (CAPTEM)

There are few randomized control trials of chemotherapy treatment in PNEN. The combination of capecitabine and temozolomide (CAPTEM) vs. temozolomide was tested in a phase II randomized controlled trial enrolling patients with advanced G1/G2 PNEN [42]. A total of 144 patients were enrolled, half treated with temozolomide (200 mg/m^2^ QD for 5 days every 28 days) and half with CAPTEM (capecitabine 750 mg/m^2^ twice daily on days 1–14 and temozolomide 200 mg/m^2^ QD on days 10–14 every 28 days) for a scheduled 13 cycles. A significantly improved PFS was reported in patients receiving CAPTEM (23 months) compared to those treated with temozolomide (14 months), in the interim but not the final analysis (HR 0.58, 95% CI, 0.36 to 0.93, *p* = 0.022). Overall survival time was also significantly prolonged in the CAPTEM vs. temozolomide arms in the interim but not in the final analysis (HR 0.41, 95% CI, 0.21 to 0.82, *p* = 0.012). The rate of treatment discontinuation due to side effects was 6% in the temozolomide arm and 15% in the CAPTEM arm, with 22% vs. 45% grade 3–4 adverse events, respectively, which mostly included neutropenia and thrombocytopenia. The most common adverse events in the CAPTEM arm were nausea (65%), fatigue (56%), constipation (48%), and anemia (37%), and in the temozolomide arm fatigue (63%), nausea (60%), constipation (31%), anemia (31%), and thrombocytopenia (31%).

## 4. Proposed Treatment Algorithm

**Figure 1:**
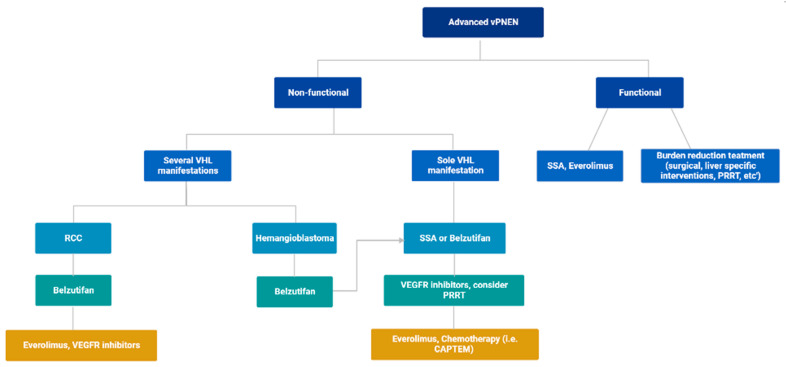
Advanced vPNEN treatment algorithm according to patient specific characteristics. Treatment decision is based on efficacy of therapy as studied in patients with vPNET followed by known efficacy regarding other clinical manifestations or functionality of the vPNEN. CAPTEM—capecitabine and temozolomide, PRRT—peptide receptor radionuclide therapy, RCC- renal cell carcinoma, SSA-somatostatin analogues, VEGFR—vascular endothelial growth factor receptor, vPNEN—VHL-related pancreatic neuroendocrine neoplasm.

As detailed above, treatment of advanced vPNET is based on few prospective clinical trials that included patients with localized disease and extrapolations from treatment modalities studied in sporadic PNENs. The randomized studies directly addressing therapeutic avenues in vPNEN include several VEGFR inhibitor types with varying efficacy and toxicity, and an HIF inhibitor (belzutifan) with a relatively improved toxicity profile. However, the rarity of advanced vPNEN limits the ability to conduct prospective clinical studies directly assessing the optimal management of these complex patients. We detail below a possible approach to the patient with advanced vPNEN. The algorithm is based on the management guidelines for sPNEN [54], amended according to the data available on the natural history of VHL-related neoplasms, pharmacological interventions for VHL-related neoplasms, and their potential toxicity. Nevertheless, our suggestion should not be considered as guidelines or evidence-based suggestions, but as a general aid for decision making in these rarely encountered patients (Figure 1). Furthermore, considering the evidence paucity, we suggest that, as a rule, follow-up and treatment decisions for patients with advanced vPNEN should be based on multidisciplinary team discussions, to enable a broad consideration of all the VHL-related clinical aspects.

Treatment toxicity is a major consideration in decision making in patients with VHL. Two parameters should be calculated into the decision making. First, the accumulating toxicity to the bone marrow [55], due to the potential need for chemotherapy later in the patient’s life course [56]. Second, in VHL there is a unique goal to enable “normal life” alongside the management of cancer, whenever feasible. Hence, high-grade adverse events should be avoided as much as possible, since patients with VHL have cancer and other neoplasms as a chronic disease [57]. 

The suggested treatment algorithm takes into consideration several possible clinical scenarios that a patient with VHL may encounter. First, patients with VHL often harbor multiple VHL-related manifestations [4]. Thus, pharmacological treatment that is ideally relevant for all current manifestations should be preferred, such as VEGFR inhibitors and belzutifan that have proven efficacy for several VHL-related neoplasms [22,24,25,58]. For example, a patient with both vPNEN and hemangioblastomas could benefit from treatment with belzutifan for both manifestations, as belzutifan is the only systemic therapy thus far that has shown beneficial results for hemangioblastomas [37] in VHL. Hence, it may be preferred as a first line treatment. Naturally, one should consider the lack of prospective data on the efficacy of belzutifan for advanced PNEN. A patient with VHL manifesting both with vPNEN and non-metastatic RCC could be treated either with belzutifan, everolimus, or a VEGFR inhibitor such as cabozantinib or sunitinib, as all three therapeutic routes have beneficial effects for PNEN and RCC [25,36,40,51,52,59]. Furthermore, since belzutifan and VEGFR inhibitors have shown efficacy against vPNET, they might be preferred compared with everolimus, which was not studied for these neoplasms [60]. Second, patients with VHL might require systemic therapy for other VHL-related manifestations in the long term, including chemotherapy, and thus interventions that affect bone marrow function, such as PRRT [5,6,61], might better be reserved as a second line treatment. Finally, in the very rare case of a functional vPNEN—insulin, gastrin, glucagon or vasoactive intestinal peptide-secreting PNEN (insulinoma, gastrinoma, glucagonoma, and VIPoma, respectively,)—the management should encompass two issues: the oncologic aspects, and the endocrine aspects. The two aspects are often co-treated when using drugs that reduce both cell proliferation and secretion (somatostatin analogues for all tumors [3,62], mTOR inhibitors for insulinoma [63,64], etc.,). However, considering tumor load reduction, not aiming for “no evidence of disease” in metastatic disease is unique for functional advanced tumors. Disease burden reduction can be achieved by surgical debulking interventions, often including hepatic metastasectomy or lobectomy; liver-directed intervention using invasive radiology techniques and injection of various compounds, such as selective internal radiation therapy (SIRT) with the beta radiation emitting Yttrium-99 (SIRT) [65] or transarterial chemoembolization (TACE) [66,67]; selective radiofrequency ablation (RFA) [54] of pancreatic lesions or hepatic metastases; and PRRT with ^177^Lu-DOTATATE [68].

## 5. Conclusions and Future Directions

In summary, pancreatic neuroendocrine neoplasms in the context of VHL have distinct characteristics compared with sporadic PNENs, including a lower rate of metastases and a lower grade, and are mostly non-functional neoplasms. The entry of belzutifan is a game changer in the management of VHL and, specifically, of vPNEN. While there is no evidence on the efficacy of belzutifan for advanced vPNEN, such data are anticipated in the coming years and, considering the supportive results on the efficacy of belzutifan for localized vPNEN, it is prudent to expect that it will become a key pharmacologic intervention for patients with advanced vPNEN. Until such evidence is available, off-label use of belzutifan may be suggested, together with the currently available interventions used for advanced sporadic PNEN.

## Figures and Tables

**Table 1:** VHL-mechanism-related treatments in randomized trials involving patients with VHL.

Treatment	Mechanism of Action	n (n vPNEN)	Efficacy n Response/Total Lesions	Toxicity %
Sunitinib [22]	VEGFR inhibitor	15 (7)	RCC 10/18 SD, 6/18 PR, 2/18 PD PNEN 5/5 SD CNS HB 19/21 SD, 2/21 PD Retinal HB 7/7 SD	Stopped treatment—40%
Vandetanib [23] ongoing, partial data	VEGFR inhibitor	37 (2)	PNEN 2/2 DS CNS HB 2/2 SD	Stopped treatment—19% (11% due to side effects)
Pazopanib [24]	VEGFR inhibitor	32 (17 *)	RCC 28/59 SD, 29/59 PR, 2/59 CR Pancreatic lesions 8/17 SD, 9/17 PR, 0/17 CR CNS HB 47/49 SD, 2/49 PR, 0/49 CR	Dose reduction—58%, Stopped treatment—23%
Belzutifan [25]	HIF 2 inhibitor	61 (22)	RCC 30/61 SD, 30/61 PR PNEN 20/22 (91%) confirmed response, 3 CR CNS HB—15/50 confirmed response, 3 CR Retinal HB—12/12 confirmed response	Dose reduction—15%, Dose interruption—43%, Stopped treatment—11%

CNS—central nervous system; CR—complete response; HB—hemangioblastoma; vPNEN—VHL-related pancreatic neuroendocrine neoplasm; PD—progressive disease; PR—partial response; RCC—renal cell carcinoma; SD—stable disease; VHL—von Hippel–Lindau; * pancreatic lesions.

**Table 2:** Non-VHL-mechanism-related treatments in randomized controlled trials involving patients with neuroendocrine neoplasms.

Trial Name	Intervention	No. Patients (Intervention/Control, n)	No. Patients with PNEN (Intervention/Control, n)	Grade 1/2/3 (n)	PFS Intervention vs. Control (Months) HR, 95% CI, *p* Value	OS Intervention vs. Control (Months) HR, 95% CI, *p* value	Toxicity (%)	Notes
PROMID [38]	Octreotide LAR vs. Placebo	42/43	0/0	81/3/1	14 vs. 6 ^€^ 0.33 (0.19–0.55, *p* < 0.001)	0.81 (0.3–2.18, *p* = 0.77)	Most frequent (% not reported)—diarrhea, flatulence, cholelithiasis.	Advanced Midgut NEN
CLARINET [39]	Lanreotide autogel vs. Placebo	101/103	42/48	138/60/0	NR vs 18 0.47 (0.3–0.73, *p* < 0.001) 0.58 (0.32–1.04) ^¥^	NR	Serious AE: 3% Common AE: diarrhea 26%, abdominal pain 14%, cholelithiasis 10%.	Advanced GEP-NEN
RADIANT-3 [40]	Everolimus vs. Placebo	207/203	207/203	341/65/na *	11.4 vs. 5.4 0.35 (0.27–0.45, *p* < 0.001)	44 vs. 37 0.94 (0.73–1.2)	Common AE: stomatitis 64%, rash 49%, diarrhea 34%, fatigue 31%, infections 23%. Grade 3/4 AE: anemia 6%, thrombocytopenia 4%, hyperglycemia 5%, stomatitis 7%, diarrhea 3%.	Advanced PNEN
NETTER-1 [41]	PRRT plus Octreotide LAR 30 mg vs. Octreotide LAR 60 mg	116/113	0/0	157/72/0	NR vs. 8.4 0.21 (0.13–0.33, *p* < 0.001)	NR 14 vs. 26 deaths, *p* < 0.01 ^#^	Common AE: nausea 59%, vomiting 47%, fatigue 40%. Grade 3/4 AE: lymphopenia 9%, vomiting 7%, nausea 4%, thrombocytopenia 2%.	Advanced midgut NEN
ECOG-ACRIN E221 [42]	CAPTEM vs. Temozolomide	72/72	72/72	50/61/na	23 vs. 14 0.58 (0.36–0.93, *p* = 0.022) ^#^	59 vs. 54 0.41 (0.21–0.82, *p* = 0.012) ^#^	Common AE: CAPTEM—nausea 65%, fatigue 56%, constipation 48%, anemia 37%. Temozolomide—fatigue 63%, nausea 60%, constipation 31%, anemia 31%, thrombocytopenia 31%.	Advanced PNEN

AE—adverse events; CAPTEM—capecitabine/temozolomide; CR—complete response; GEP—gastro-entero-pancreatic; na—not available; NEN—neuroendocrine neoplasm; NR—not reached; PRRT—peptide receptor radionuclide therapy; VHL—von Hippel–Lindau; * grade is defined as well- and moderately differentiated NEN in this trial without report on definition or grade; ^#^ interim analysis; ^¥^ subgroup analysis including PNEN only; ^€^ The PROMID study reported time to progression.
